# Ongoing selection for a uniquely human null allele of *SIGLEC12* in world‐wide populations may protect against the risk of advanced carcinomas

**DOI:** 10.1096/fba.2021-00036

**Published:** 2021-03-30

**Authors:** Shoib S. Siddiqui, Michael Vaill, Ajit Varki

**Affiliations:** ^1^ Departments of Medicine and Cellular and Molecular Medicine Glycobiology Research and Training Center, and Center for Academic Research and Training in Anthropogeny University of California San Diego CA USA; ^2^ Present address: School of Life and Medical Sciences University of Hertfordshire Hatfield UK

The CD33‐related subset of Siglecs (Sialic Acid binding Immunoglobulin superfamily Lectins) are well‐known signaling receptors on immune cells, but Siglec‐XII is also expressed on epithelial cell surfaces.^1^, ^2^ A point mutation eliminating the canonical sialic acid‐binding function of Siglec‐XII is unique to humans^2^, ^3^ and fixed in all populations worldwide. We and others reported a further polymorphic frameshift mutation in the human *SIGLEC12* gene that appears to be undergoing selection favoring a null and/or truncated allele in all human populations.^1^, ^4^, ^5^ Individuals who have an ancestral *SIGLEC12* allele with an intact open reading frame appear more prone to develop advanced carcinomas.^6^ The likely mechanism involves aberrant signaling via recruitment of the SHP2 phosphatase to the cytosolic tail of the dysfunctional Siglec‐XII.

We appreciate the strongly supportive and positive comments by Voskarides^7^ about our article published in this journal,^6^ which suggested that human‐specific polymorphic pseudogenization of *SIGLEC12* protects against advanced cancer progression. Voskarides appropriately emphasizes the relevance of George Williams' classic theory of “antagonistic pleiotropy”,^8^ in which selection for an allele's beneficial effects in early life can have deleterious consequences in late life.^9^ A 2018 meta‐analysis of 247 Genome‐Wide Association Studies (GWAS) identified an association between 1377 cancer‐associated genes in populations living in extremely cold environments and very high altitudes.^10^, ^11^, ^12^ It is suggested that genetic variants that contributed to the survival of early humans living in these extreme environments are now associated with cancer incidence in contemporary populations, that is, the accumulation of deleterious mutations in tumor‐suppressor, apoptosis, and cell cycle regulation genes.^7^

In response to the interesting commentary which compares our discovery with previous examples of antagonistic pleiotropy, we should emphasize that our findings on Siglec‐XII are somewhat different from these instances. First, we report selection for the null alleles of *SIGLEC12* in all human populations. Second, *SIGLEC12* expression is not associated with the incidence or risk for carcinomas. Rather, the null state is associated with a decrease in advanced carcinomas commonly found in humans.^1^, ^6^, ^13^ Third, unlike the genes discussed by Voskarides, many of which are extensively studied and fundamental to cancer biology, Siglec‐XII has received very little attention to date. Moreover, our studies represent the first implications of *SIGLEC12* in human cancer, with currently unknown potential impact on cancer biology, prevention, and therapy.

Despite the above differences between our findings^6^ and those of Voskarides, we believe his perspective gives some useful additional clues. First, the deleterious *SIGLEC12* mutation is not fully studied and understood under stressful conditions. It might be that the status of *SIGLEC12* in populations living in extreme vs non‐extreme conditions is different, following the trend shown for tumor‐suppressor and DNA repair genes. Our data also shows that unlike the other examples, Siglec‐XII seems to be a suitable prognostic marker. In a large cohort study of late‐stage colon cancer patients, we found that Siglec‐XII non‐expressors had a two fold higher survival than expressors.^6^ Siglec‐XII also has the potential to be a therapeutic target, as we previously also showed that it undergoes rapid endocytosis upon binding a Siglec‐XII antibody, allowing selective delivery of a conjugated toxin.^1^

In keeping with the conventional theory, Voskarides also emphasizes that it is very hard for natural selection to eliminate mutations that cause cancer after the end of reproductive age. With *SIGLEC12*, ongoing selection for the null state might be an example of a “less‐is‐more” evolutionary scenario, where losing expression of the full‐length protein is beneficial under current selective pressures.^6^, ^14^, ^15^ It is also potentially an example of selection for prolonged post‐reproductive life span, a trait that is unique to humans among all land mammals. Indeed, it is consistent with the grandmother hypothesis, which posits that post‐reproductive individuals (predominantly grandmothers) can improve the survival of helpless younger kin.^16^, ^17^, ^18^ In this regard, we have reported one example of a uniquely human allele of *CD33* that protects against cognitive decline during the post‐reproductive lifespan of humans.^19^, ^20^ Overall, we believe that the scenario with Siglec‐XII is different from other cancer genes undergoing positive selection, but further studies are needed.
